# TMEM16F/Anoctamin 6 in Ferroptotic Cell Death

**DOI:** 10.3390/cancers11050625

**Published:** 2019-05-05

**Authors:** Jiraporn Ousingsawat, Rainer Schreiber, Karl Kunzelmann

**Affiliations:** Institut für Physiologie, Universität Regensburg, Universitätsstraße 31, D-93053 Regensburg, Germany; Jiraporn.Ousingsawat@vkl.uni-regensburg.de (J.O.); Rainer.Schreiber@vkl.uni-regensburg.de (R.S.)

**Keywords:** Anoctamin 6, TMEM16F, apoptosis, ferroptosis, Ca^2+^ signaling, cell death

## Abstract

Ca^2+^ activated Cl^−^ channels (TMEM16A; ANO1) support cell proliferation and cancer growth. Expression of TMEM16A is strongly enhanced in different types of malignomas. In contrast, TMEM16F (ANO6) operates as a Ca^2+^ activated chloride/nonselective ion channel and scrambles membrane phospholipids to expose phosphatidylserine at the cell surface. Both phospholipid scrambling and cell swelling induced through activation of nonselective ion currents appear to destabilize the plasma membrane thereby causing cell death. There is growing evidence that activation of TMEM16F contributes to various forms of regulated cell death. In the present study, we demonstrate that ferroptotic cell death, occurring during peroxidation of plasma membrane phospholipids activates TMEM16F. Ferroptosis was induced by erastin, an inhibitor of the cystine-glutamate antiporter and RSL3, an inhibitor of glutathione peroxidase 4 (GPX4). Cell death was largely reduced in the intestinal epithelium, and in peritoneal macrophages isolated from mice with tissue-specific knockout of TMEM16F. We show that TMEM16F is activated during erastin and RSL3-induced ferroptosis. In contrast, inhibition of ferroptosis by ferrostatin-1 and by inhibitors of TMEM16F block TMEM16F currents and inhibit cell death. We conclude that activation of TMEM16F is a crucial component during ferroptotic cell death, a finding that may be useful to induce cell death in cancer cells.

## 1. Introduction

TMEM16A-K (anoctamin 1–10) form a family of 10 paralogous proteins that are Ca^2+^ activated phospholipid scramblases and ion channels [1,2,3]. These proteins are broadly expressed and fulfill numerous functions in epithelial cells and other non-excitable tissues, as well neurons, smooth muscles and sensory cells. The Ca^2+^ activated Cl^−^ channel TMEM16A has been analyzed in great detail, and has been found along with other members of the TMEM16 family to control cell proliferation and growth of different types of cancer (reviewed in [4,5]. Thus, inhibition of TMEM16A is a novel way to interfere with cell proliferation and growth of cancer [5,6,7,8,9].

In contrast, TMEM16F functions as Ca^2+^-activated phospholipid scramblase [10,11,12], while others highlighted the Ca^2+^ permeability of TMEM16F [13]. Most reports confirmed a Cl^−^ permeability for TMEM16F [14,15,16,17], while others reported non-selectivity for TMEM16F [18]. We found that the TMEM16F produces Cl^−^ selective currents at intracellular Ca^2+^ concentrations in the low µmolar range, while ongoing stimulation and higher intracellular Ca^2+^ concentrations increase non-selectivity of the channel [19]. Moreover, TMEM16F has a role in macrophage and lymphocyte death, caused by massive P2X_7_-induced increase in intracellular Ca^2+^ [15,19]. TMEM16F is also present in pre-apoptotic cells of the intestinal surface epithelium, but not in proliferative intestinal crypt cells [15]. Subsequent studies suggested a contribution of TMEM16F to different forms of regulated cell death. Pyroptosis, for example, is a highly inflammatory form of programmed cell death caused by intracellular pathogens and activation of inflammasomes [20,21]. Gasdermin D generates large plasma membrane pores, a process that depends on expression of TMEM16F [22]. Remarkably, lipid peroxidation activates TMEM16F [23] and drives pyroptosis [24]. Pore formation and cell death due to pulsed electric fields also requires activation of TMEM16F [25]. Another report demonstrated activation of TMEM16F during necroptotic cell death, although it does not seem to be essential for necroptosis [26]. TMEM16F is normally present in the so-called primary cilium of renal collecting duct epithelial cells. It is, however, highly upregulated in pre-apoptotic epithelial cells in the center of growing renal cysts, where it supports cyst expansion in polycystic kidney disease [27].

Recent data indicate that TMEM16F is also activated by an increase in reactive oxygen species (ROS) and lipid peroxidation [28], as well as phospholipid hydrolysis caused by phospholipase A2 [23]. We therefore examined in the present study the role of TMEM16F during ferroptosis, a form of regulated cell death that occurs due to accumulation of lethal phospholipid peroxides [29]. The present data obtained from mice lacking expression of TMEM16F and from TMEM16F-knockout cells in vitro, strongly suggest that elimination of TMEM16F-expression or inhibition TMEM16F protects from ferroptotic cell death. Activation of TMEM16F may therefore enhance ferroptotic cell death in cancer cells.

## 2. Results

### 2.1. Attenuated Cell Death in Epithelial Cells from Mice Lacking Expression of TMEM16F

**Figure 1:**
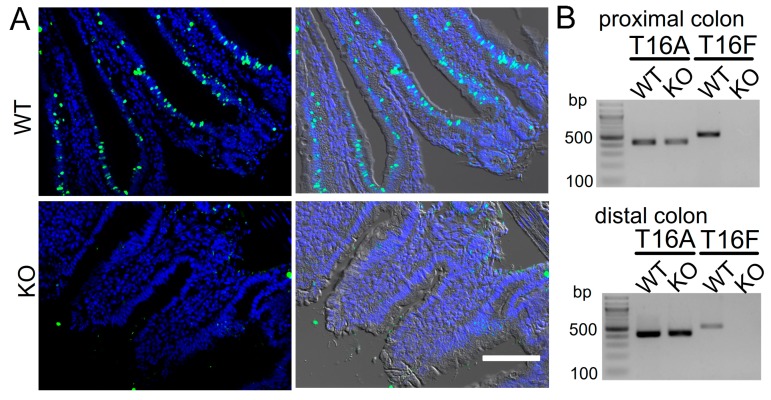

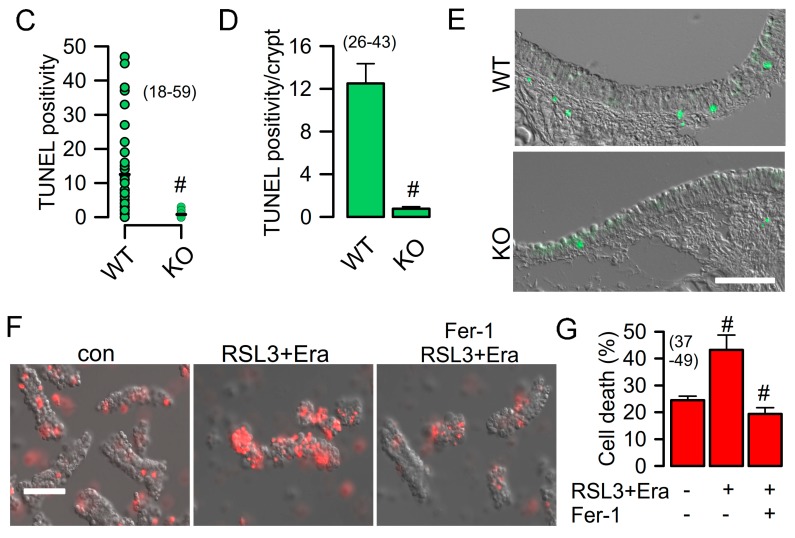
Attenuated cell death in epithelial cells from mice lacking expression of TMEM16F. (**A**) TUNEL staining in intestinal epithelium of mice with intestinal epithelial specific knockout of TMEM16F (TMEM16F^fl/fl^-Vil1-Cre; KO) and WT littermate controls (TMEM16F^fl/fl^; WT). Bar = 100 µm. (**B**) RT-PCR indicating lack of expression of TMEM16F in isolated crypts of TMEM16F knockout animals. (**C**,**D**) Summary of the number of TUNEL-positive crypts per section and the number of TUNEL-positive cells per individual crypt, respectively. (**E**) TUNEL positive cells in airways of WT mice and mice with airway epithelial specific knockout of TMEM16F [31]. Bar = 50 µm. (**F**,**G**) Propidium iodide staining in isolated mouse crypts and activation of ferroptosis by RSL3 (1 µM) and erastin (5 µM) (RSL3+Era). Inhibition of ferroptotic cell death by ferrostatin-1 (Fer-1; 5 µM). Bar = 100 µm. Mean ± SEM (number of experiments). ^#^ significant difference compared to WT, con, or RSL3+Era, respectively (*p* < 0.05, unpaired *t*-test).

We reported earlier a pro-apoptotic effect of TMEM16F in macrophages [19]. TMEM16F is expressed in the intestinal surface epithelium, but not in intestinal crypts [15]. Stem cells in the crypt base proliferate, while aged enterocytes in the surface epithelium are prone to cell death. These dying cells are replaced by cells from the lower crypt, which move upwards and towards the surface epithelium [30]. We generated mice with an intestinal epithelial specific knockout of TMEM16F [31]. TUNEL assays performed in intestinal epithelium of TMEM16F knockout mice unmasked an impressive inhibition of spontaneous cell death, when compared to wild type mice (Figure 1A–D). In contrast to intestinal cells, airway epithelial cells are replaced at a much lower rate. Although we did not examine airways systematically, airway epithelial-specific knockout of TMEM16F [31] also seemed to reduce cell death in the airway epithelium (Figure 1E). We isolated intestinal crypts, which typically move into anoikis, another form of programmed cell death caused by a loss of contact with the basolateral matrix [32] (Figure 1F). Ferrostatin-1, an inhibitor of ferroptosis that was also shown to inhibit activation of TMEM16A/F [33], did not suppress anoikis but inhibited ferroptotic cell death induced by RSL3 and erastin (Era) [34] (Figure 1F,G).

### 2.2. Reduced Ferroptosis in Macrophages from Mice Lacking Expression of TMEM16F

**Figure 2:**
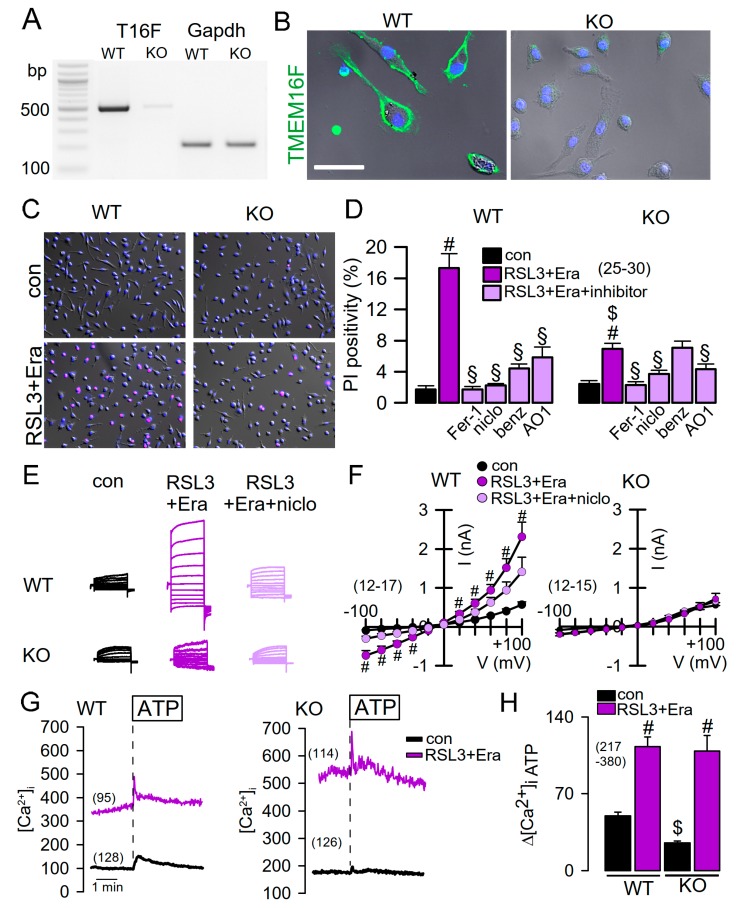
Reduced ferroptosis in macrophages from mice lacking expression of TMEM16F. (**A**) Semiquantitative RT-PCR indicating lack of expression of TMEM16F (T16F) in peritoneal macrophages isolated from mice with a tissue specific knockout of TMEM16F (TMEM16F^flox/flox^-Cx3cr1-Cre, KO; c.f. Methods). (**B**) Immunostaining of TMEM16F (green) in WT but not KO macrophages. Nuclei labeling by DAPI (blue). Bar = 20 µm. (**C**,**D**) Propidium iodide (PI) staining of WT and KO macrophages and induction by RSL3 + erastin (1/5 µM; RSL3-Era). Inhibition of PI positivity by ferrostatin-1 (Fer-1; 5 µM) and by inhibitors of TMEM16F, niclosamide (niclo; 1 µM), benzbromarone (benz; 5 µM), and CaCCinhAO1 (AO1; 10 µM), respectively. (**E**) Activation of whole cell currents by RSL3+Era in WT but not KO macrophages, and inhibition of current activation by niclosamide (1 µM). (**F**) Corresponding current/voltage relationships of currents measured in WT and KO macrophages. (**G**,**H**) Measurement of intracellular Ca^2+^ concentrations before (con, black line) and after incubation with RSL3 and erastin (RSL3+Era, purple), and activation of Ca^2+^ store release by ATP (100 µM). RSL3+Era significantly increase basal intracellular Ca^2+^ levels and augment ATP-induced Ca^2+^ store release. Mean ± SEM (number of experiments). ^#^ significant difference when compared with control (*p* < 0.05, ANOVA). ^$^ significant difference when compared with WT (*p* < 0.05, ANOVA). ^§^ significant inhibition by blockers (*p* < 0.05, ANOVA).

To further examine a potential role of TMEM16F for ferroptosis, we used Cx3cr1-Cre mice to generate a knockout of TMEM16F in macrophages (c.f. Methods) (Figure 2A,B). Macrophages express TMEM16F at relatively high levels, which has a significant impact on essential macrophage functions, as shown earlier in conventional TMEM16F knockout macrophages [19]. We induced ferroptotic cell death by RSL3 and erastin in isolated peritoneal macrophages. Cell death was detected by propidium iodide (PI) staining. Knockout of TMEM16F largely reduced ferroptosis in macrophages (Figure 2C,D). Moreover, ferrostatin-1 and different inhibitors of TMEM16F, such as niclosamide, benzbromarone, or CaCCinhAO1 strongly inhibited ferroptosis (Figire 2D). In patch clamp experiments, a whole cell current was activated by RSL3/erastin in WT macrophages, which was absent in KO macrophages (Figure 2E,F). Measurement of intracellular Ca^2+^ concentrations indicated a sharp increase in basal intracellular Ca^2+^ levels during ferroptosis, which explains the spontaneous activity of TMEM16F currents in WT macrophages (Figure 2G,H). Moreover, release of Ca^2+^ from intracellular endoplasmic reticulum Ca^2+^ stores was augmented after RSL3/erastin (Figure 2G,H).

**Figure 3:**
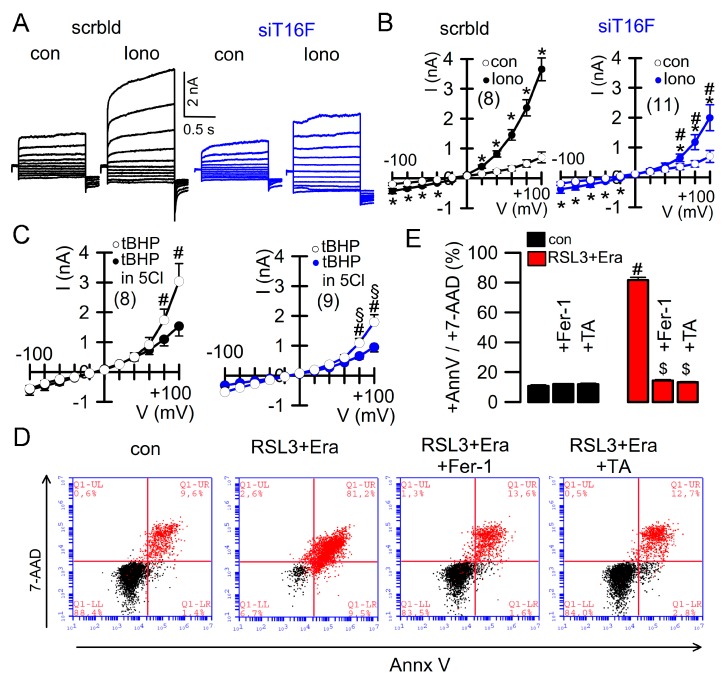
Activation of cell death in in Jurkat T-lymphocytes. (**A**,**B**) Whole cell patch clamp currents activated by ionomycin (Iono, 1 µM) in Jurkat T lymphocytes, and corresponding current/voltage relationships. (**C**) Activation of whole cell currents by *tert*-butyl hydroperoxide (tBHP; 50 μM/6 h) and inhibition of currents by 5Cl. (**D**,**E**) Activation of cell death by RSL3 + erastin and inhibition by Figure 1. (Fer-1, 5 µM) or tannic acid (TA, 10 µM). Mean ± SEM (number of experiments). * significant activation by Iono (*p* < 0.05, paired *t*-test). ^#^ significant difference when compared with scrambled, control, or 5Cl, respectively (*p* < 0.05, unpaired *t*-test). ^$^ significant inhibition (*p* < 0.05, ANOVA).

Similar to macrophages, Jurkat T-lymphocytes also express TMEM16F, which can be activated by increase in intracellular Ca^2+^ using the Ca^2+^ ionophore ionomycin (Iono) or by lipid-peroxidizing tert-butyl hydroperoxide (tBHP) [15,23] (Figure 3A–C). Activation of TMEM16F currents was inhibited by siRNA-knockdown of TMEM16F-expression (siTMEM16F). Moreover, the pronounced ferroptotic cell death induced by RSL3/erastin was remarkably blocked by ferrostatin-1 and by the inhibitor of TMEM16F, tannic acid [35] (TA; Figure 3D,E). These results demonstrate a significant contribution of endogenous TMEM16F to ferroptosis.

### 2.3. Effect of Overexpressed TMEM16F and Cooperativity with TMEM16A

**Figure 4:**
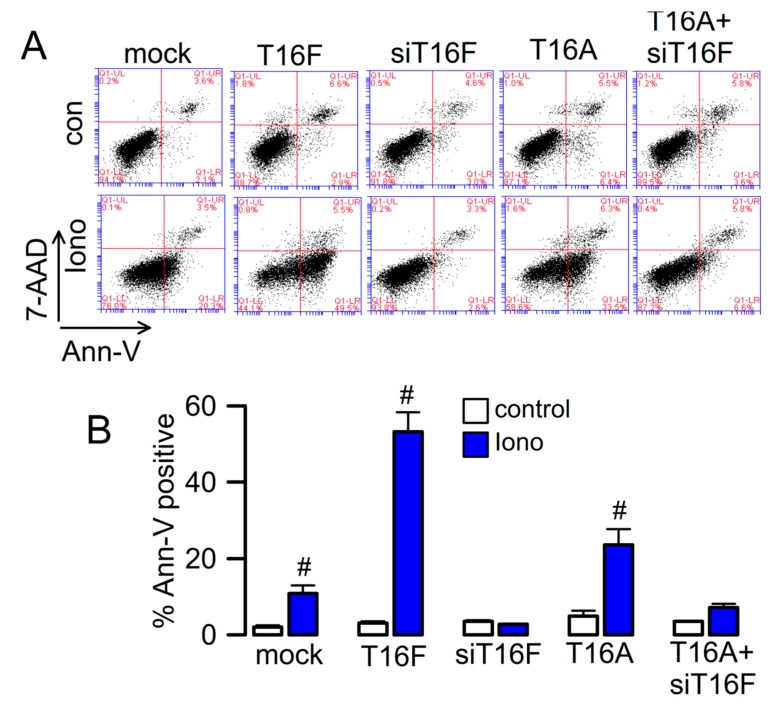
Phospholipid scrambling by TMEM16F and cooperativity with TMEM16A. (**A**) Flow cytometry in HEK293 cells expressing TMEM16F, TMEM16A, TMEM16A in the presence of siRNA for TMEM16F, or cells transfected with empty plasmid (mock) or siRNA for TMEM16F. 4-quadrant dot blot graphs showing 7-AAD positivity on y-axis and annexin V positivity on x-axis. Phospholipid scrambling (PS; annexin V positivity) was induced by stimulation of the cells with 1 µM Iono; 10 min). (**B**) Summary of % annexin V positive cells before and after exposure to ionomycin. Mean ± SEM (number of experiments). ^#^ indicates significant difference when compared to control (*p* < 0.05, unpaired *t*-test).

Overexpressed TMEM16F scrambles plasma membrane phospholipids and conducts ions [36]. We overexpressed TMEM16F in HEK293 cells and analyzed exposure of phosphatidylserine (PS; annexing V binding) by flow cytometry, upon stimulation with ionomycin (Figure 4A,B). We detected low but detectable scrambling activity also in mock transfected cells, which was due to endogenous expression of TMEM16F, as demonstrated by siRNA-knockout of TMEM16F. Interestingly, TMEM16A also augmented PS exposure, which was however, abolished by simultaneous knockdown of TMEM16F. Because TMEM16A does not scramble phospholipids, but augments Ca^2+^ store release and consecutive store operated Ca^2+^ entry (SOCE) [37,38], we suggest that TMEM16A induces PS exposure indirectly by activation of SOCE that provides Ca^2+^ for activation of TMEM16F [38].

### 2.4. Ferroptosis Induced in Cancer Cells

**Figure 5:**
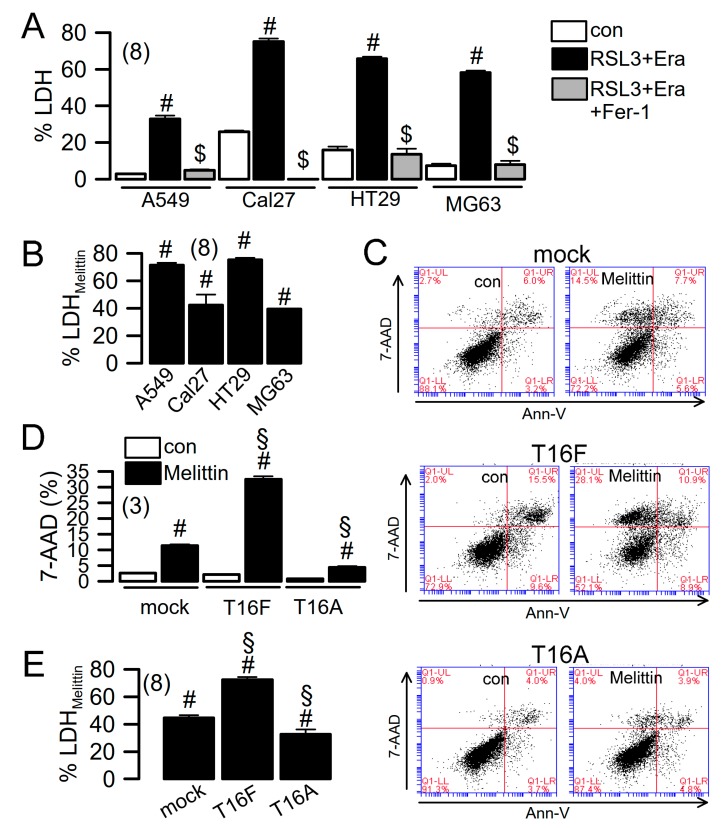
Cell death induced in cancer cells. (**A**) Summary of LDH release induced by RSL3 + Era (24 h) in human A549 pulmonary adenocarcinoma, Cal27 head and neck cancer, HT_29_ colonic carcinoma, and MG-63 osteosarcoma cells, respectively. (**B**) Summary of LDH release induced in cancer cells by melittin (1 µM/14 h). (**C**,**D**) Dot blot indicating cell death induced by melittin in HEK293 cells expressing empty plasmid, TMEM16F, or TMEM16A, respectively. 7-AAD positivity indicating enhanced cell death in cells expressing TMEM16F. (**E**) Cell death induced by melittin, as detected by LDH release. Mean ± SEM (number of experiments). ^#^ Significant induction of LDH release or 7-AAD positivity, respectively (*p* < 0.05, unpaired t-test). ^$^ Significant inhibition by Fer-1 (*p* < 0.05, unpaired *t*-test). ^§^ Significant difference compared to mock (*p* < 0.05, ANOVA).

**Figure 6:**
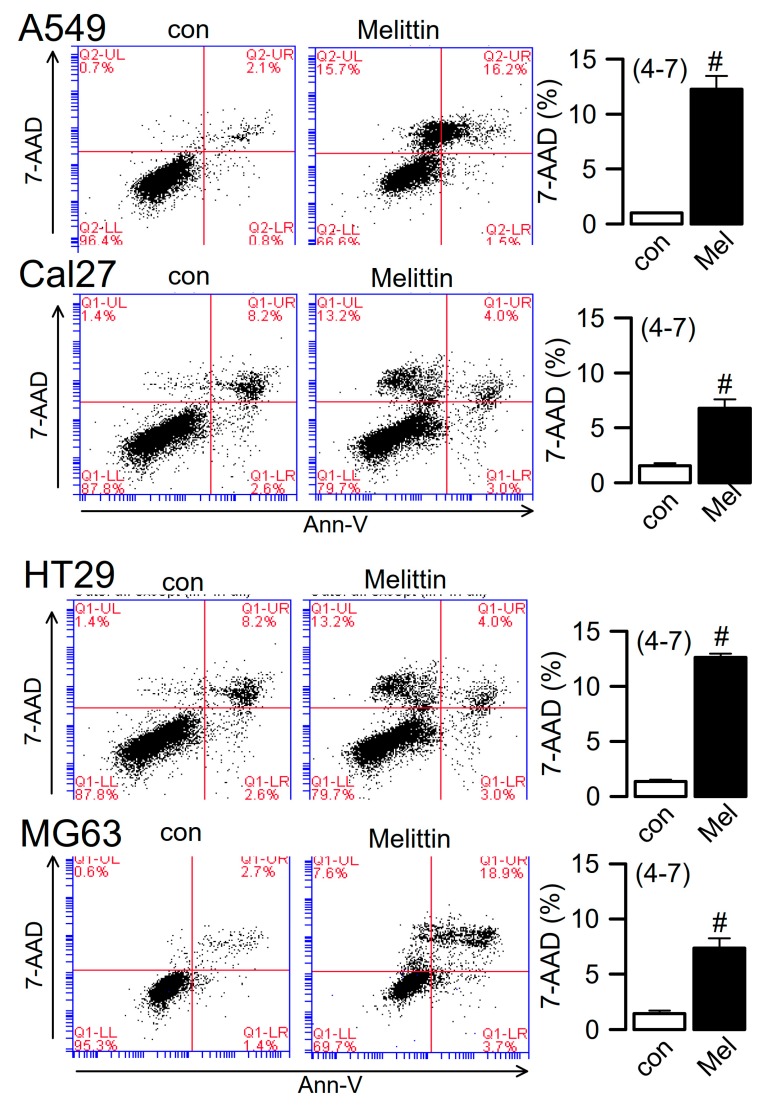
Melittin activated cell death of cancer cells. Dot blots indicating cell death (7-AAD positivity) induced by melittin (1 µM/14 h) in A549, Cal27, HT_29_ and MG-63 cells. Summary of 7-AAD positivity before and after application of melittin. Mean ± SEM (number of experiments). ^#^ Significant difference when compared to control (*p* < 0.05, unpaired *t*-test).

TMEM16F is widely expressed in different cell types, including macrophages and lymphocytes, pre-apoptotic cells, and cancer cells [26,39]. By measuring LDH release, we found that ferroptotic cell death is induced in human A549 pulmonary adenocarcinoma, Cal27 head and neck cancer, HT_29_ colonic carcinoma, and MG-63 osteosarcoma cells, respectively. LDH release was completely inhibited by ferrostatin-1 (Figure 5A). Destabilization of the plasma membrane is an essential feature of lipid peroxidation/ferroptotic cell death, which also occurs during activation of phospholipase A2 (PLA2) [29,35]. Both lipid peroxidation and PLA2 activates TMEM16F [23]. We found that similar to RSL3/erastin, the PLA2-activator melittin induced release of LDH (Figure 5B) and uptake of 7-AAD (a replacement for propidium iodide), as detected by flow cytometry (Figure 6). Overexpression of TMEM16 proteins in HEK293 cells confirms that activation of TMEM16F, but not TMEM16A, is responsible for melittin-induced cell death. Thus, TMEM16F is an essential factor that contributes to membrane destabilization during induction of ferroptotic cell death, probably due to its ability to scramble membrane phospholipids. Direct activation of TMEM16F may therefore resemble a novel strategy for induction of cell death in cancer cells.

## 3. Discussion

TMEM16F is broadly expressed in all types of tissues, albeit at very different levels. It appears to be upregulated in cells undergoing regulated cell death [15,27]. Here, we provide evidence that TMEM16F is activated during ferroptosis, a regulated cell death pathway triggered by oxidation of preferentially polyunsaturated fatty acid (PUFA)-containing membrane phospholipids [29]. Ferroptosis is induced experimentally using erastin, an inhibitor of the cystine-glutamate antiporter System Xc-(*SLC7A11).* Cystine import into cells is required to produce glutathione (GSH) used by glutathione peroxidase 4 (GPX4) to eliminate lipid peroxides. RSL3, an inhibitor of GPX4, further augments ferroptosis. Ferroptotic cell death may trigger sterile inflammation through the release of danger-associated molecular patterns (DAMP), recognized by innate immune receptors. Thus, ferroptotic cell death and TLR4-dependent signaling in graft endothelial cells is causing tissue inflammation after cardiac transplantation [40]. Similarly, acute kidney injury and synchronized renal tubular cell death involves ferroptosis [41]. 

We showed earlier that ROS induced lipid peroxidation activates TMEM16F (and TMEM16A) in a Ca^2+^ independent fashion. Moreover, plasma membrane lipid modifications through other pathways such as phospholipase A2 (PLA2) also activate both TMEM16A and TMEM16F [23]. The lipid-dependence of TMEM16 proteins may not be surprising given the intimacy between the TMEM16 pore and membrane phospholipids [12,42,43,44]. The structure of TMEM16F is highly dynamic, with an equilibrium between different states. This suggests additional factors apart from calcium, which may tightly lock the pore in either a closed state, an intermediate state allowing transport of ions, or a phospholipid transporting state [42,45]. Recent structural analysis of TMEM16F may also provide an explanation for the differences between overexpressed and endogenous TMEM16, and their temperature-dependence [23,46,47].

TMEM16F scrambles phospholipids and conducts cations and anions. We found earlier that ongoing stimulation by the Ca^2+^ ionophore ionomycin induces non-selectivity of the ion current produced by TMEM16F [19]. A nonselective TMEM16F current will depolarize the membrane voltage and lead to cell swelling. Cell swelling in conjunction with phospholipid scrambling, membrane blebbing and destabilization of the plasma membrane, explains cell death elicited by activation of TMEM16F [19,25,48,49]. Interestingly, lipid peroxidation drives gasdermin D-mediated pyroptosis in lethal polymicrobial sepsis [24]. This may explain why we also found a role of TMEM16F in pyroptosis [22].

Induction of ferroptotic cell death is considered a novel pathway to eliminate cancer cells [50,51]. This is achieved by using agents that increase ROS generation, or by compounds that inhibit antioxidant defense [28]. Some of these compounds have already entered clinical trials. They can kill cancer cells effectively and can antagonize the development of drug resistance. Because the present data demonstrate a central role of TMEM16F during ferroptotic cell death, we propose direct activation of TMEM16F as a promising new strategy to interfere with cancer growth.

**Figure 7:**
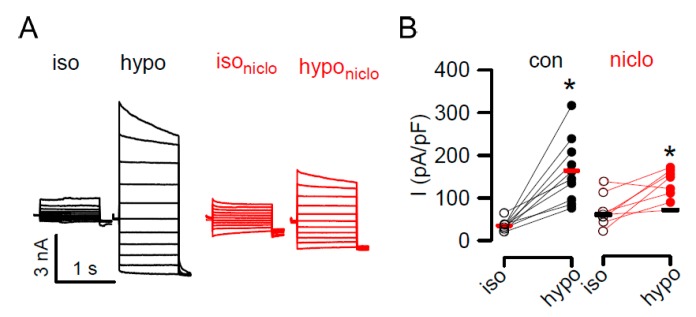
Activation of VRAC is inhibited by niclosamide. (**A**,**B**) Whole cell patch clamp currents activated by hypotonic cell swelling (hypo; 33% hypotonicity) in Jurkat T lymphocytes, and inhibition of VRAC activation by niclosamide (niclo; 1 µM). Summary of single experiments and mean ± SEM indicating significant inhibition of VRAC by niclosamide (*p* < 0.05, unpaired *t*-test). * significant activation by (*p* < 0.05, paired *t*-test).

TMEM16F supports Ca^2+^ activated membrane trafficking and controls membrane exocytosis [52,53]. Possible underlying mechanisms have been discussed recently [54]. Along this line, membrane exposure of LRRC8A, the essential subunit of the volume regulated anion channel (VRAC), depends on activation of TMEM16A [55]. VRAC controls cell death and chemoresistance of cancer cells, and is therefore of relevance for tumor biology [56,57]. As shown in Figure 4, activation of TMEM16A supports phospholipid scrambling by activating TMEM16F and therefore supports activation of VRAC [58]. Thus, it is not surprising that the potent inhibitor of TMEM16A, niclosamide, also inhibits activation of VRAC [59] (Figure 7). Taken together, while TMEM16A supports growth of cancer, activation of TMEM16F instead promotes cell death [60]. As there are currently no specific activators or inhibitors available for the different TMEM16 paralogues, it will be necessary to search for novel small molecule compounds that specifically activate TMEM16F, to be used in the future treatment of cancer. 

## 4. Methods

### 4.1. Immunocytochemistry

Cells were fixed for 10 min with 4% (w/v) paraformaldehyde at room temperature. After washing, cells were permeabilized with 0.5% (v/v, PBS) Triton X-100 for 10 min and blocked with 1% (w/v, PBS) bovine serum albumin for 1 h at room temperature. Cells were incubated for 1 h with primary antibodies (1:300) against TMEM16F (Davids Biotechnology, Regensburg, Germany). Binding of the primary antibody was visualized by incubation with appropriate secondary antibodies conjugated with AlexaFluor 488 (1:500, Molecular Probes, Invitrogen). Nuclei were stained with Hoe33342 (0.1 g/mL PBS, Applichem, Darmstadt, Germany) or DAPI. Glass cover slips were mounted on glass slides with fluorescent mounting medium (DAKO Cytomation, Hamburg, Germany) and examined with an ApoTome Axiovert 200 M fluorescence microscope (Zeiss, Germany).

### 4.2. Isolation of Total RNA and RT-PCR

For semi-quantitative RT-PCR of TMEM16F and TMEM16A, mRNA expression in proximal and distal segments of the mouse colonic epithelium, total RNA was isolated using NucleoSpin RNA II columns (Macherey-Nagel, Düren, Germany). Total RNA (1 µg/50 µL reaction) was reverse-transcribed using random primer (Promega, Mannheim, Germany) and M-MLV reverse transcriptase RNase H Minus (Promega, Mannheim, Germany). Each RT-PCR reaction contained sense and antisense primer for mouse TMEM16A and TMEM16F [39], 0.5 µL cDNA and GoTaq Polymerase (Promega, Mannheim, Germany). After 2 min at 95 °C cDNA was amplified 25 cycles for 30 s at 95 °C, 30 s at 56 °C and 1 min at 72 °C. PCR products were visualized by loading on Midori Green Xtra (NIPPON Genetics, Dueren, Germany) containing agarose gels and analyzed using ImageJ (NIH, Bethesda, MA, USA).

### 4.3. Calcium Measurements

Cells were seeded on glass cover slips and loaded with 2 µM Fura-2/AM and 0.02% Pluronic F-127 (Invitrogen, Darmstadt, Germany) in ringer solution (mmol/L: NaCl 145; KH_2_PO_4_ 0,4; K_2_HPO_4_ 1,6; Glucose 5; MgCl_2_ 1; Ca^2+^-Gluconat 1,3) for 1 h at room temperature. Fluorescence was detected in cells perfused with Ringer’s solution at 37 °C using an inverted microscope (Axiovert S100, Zeiss, Germany) and a high speed polychromator system (VisiChrome, Puchheim, Germany). Fura-2 was excited at 340/380 nm, and emission was recorded between 470 nm and 550 nm using a CoolSnap camera (CoolSnap HQ, Visitron, Puchheim, Germany). [Ca^2+^]*_i_* was calculated from the 340/380 nm fluorescence ratio after background subtraction. The formula used to calculate [Ca^2+^]*_i_* was [Ca^2+^]*_i_* = *Kd* × (*R* − *R*_min_)/(*R*_max_ − *R*) × (S_f2_/S_b2_), where *R* is the observed fluorescence ratio. The values *R*_max_ and *R*_min_ (maximum and minimum ratios) and the constant S_f2_/S_b2_ (fluorescence of free and Ca^2+^-bound Fura-2 at 380 nm) were calculated using 1 µM ionomycin (Calbiochem), 5 µM nigericin, 10 µM monensin (Sigma, Taufkirchen, Germany), and 5 mM EGTA to equilibrate intracellular and extracellular Ca^2+^ in intact Fura-2-loaded cells. The dissociation constant for the Fura-2•Ca^2+^ complex was taken as 224 nmol/L. Control of experiments, imaging acquisition, and data analysis were done with the software package Meta-Fluor (Molecular Devices, Biberach, Germany) and Origin (OriginLab Corporation, Northampton, MA, USA).

### 4.4. Patch Clamping

Cells were seeded on fibronectin-coated glass-coated cover slips and were mounted on the stage of an inverted microscope (Axiovert, Zeiss, Munich, Germany). Patch pipettes were filled with a cytosolic-like solution containing 30 mM KCl, 95 mM K-gluconate, 1.2 mM NaH2PO4, 4.8 mM Na_2_HPO_4_, 1 mM EGTA, 0.758 mM Ca-gluconate, 1.03 mM MgCl2, 5 mM D-glucose, and 3 mM ATP, pH 7.2. The intracellular (pipette) Ca^2+^ activity was 0.1 mM. Experiments were performed in the fast whole-cell current recordings mode. The bath was perfused continuously with Ringer solution at a rate of 8 mL/min. Patch pipettes had an input resistance of 2–4 MΩ, and whole-cell currents were corrected for serial resistance. Currents were recorded using patch clamp amplifiers (EPC 7 or EPC10; List Medical Electronics, Darmstadt, Germany), the LIH1600 interface, and PULSE software (HEKA, Lambrecht, Germany) as well as Chart software (AD Instruments, Spechbach, Germany). Cell were kept most of the time under current clamp. In regular intervals, membrane voltage (Vc) was clamped in steps of 20 mV from −100 to +100 mV from a holding voltage of −100 mV. Current densities were calculated by dividing whole-cell currents by cell capacitance.

### 4.5. TUNEL Assay

Terminal deoxynucleotidyl transferase dUTP nick end labeling (TUNEL) in intestine from mice with intestinal epithelial specific knockout of TMEM16F (TMEM16F^fl/fl^-Vil1-Cre) and WT littermate controls (TMEM16F^fl/fl^) was performed in PFA fixed tissue, embedded in paraffin. For TUNEL assay, the DeadEnd Fluorometrie TUNEL system (Promega, Mannheim, Germany) was used according to manufacturer’s instructions.

### 4.6. Flow Cytometry

Cells were collected using accutase (Capricorn Scientific, Ebsdorfergrund, Germany), washed with cold Dulbecco’s PBS (DPBS) and centrifuged at 500 g and 4 °C for 10 min. Subsequently, cells were resuspended in 100 μL annexin binding buffer containing 5 μL annexin V-FITC and 2.5 μL 7-aminoactinomycin D (7-AAD; BioLegend, Koblenz, Germany). Cells were incubated with 1 μM ionomycin for 10 min or erastin (5 µM)/RSL3 (0.1 µM) (6 h), or melittin (0.5 µM; 14 h), respectively. Reactions were stopped by adding 400 μL DPBS and cells were analysed immediately. Fluorescence activated cell sorting (FACS) analyses was performed in Annexin V standard binding buffer (BioLegend, San Diego, CA, USA) containing 10 mM Hepes, 140 mM NaCl and 2.5 mM CaCl. For each measurement, at least 10,000 cells were analysed by flow cytometry at 37 °C (BD AccuriTM C6, St. Ives, UK) 7-AAD, a non-permeant dye, was used to identify cells with plasma membrane leakage. Freshly isolated macrophages were stained with propidium iodide staining to detect RSL3/erastin-induced cell death. 

### 4.7. LDH Assay

Supernatants from 8 × 10^4^ treated cells were collected from non-treated cells, cells treated with erastin/RSL3 (5 µM)/0.1 µM; 6 h) or erastin/RSL3 + ferrostain-1 (5 µM) (all 6 h) and were measured using the CytoTox96^®^ non-radioactive cytotoxicity assay (Promega) at a wavelength of 490 nm. Percentage of LDH release was calculated as 100 × (experimental LDH-spontaneous LDH)/(maximum LDH release-spontaneous LDH).

### 4.8. Knockout Animals

TMEM16F was knocked-out in macrophages by crossbreeding with Cx3cr1-Cre mice (TMEM16F^fl/fl^-Cx3cr1-Cre). Knockout of TMEM16F expression in intestinal epithelial cells was achieved by crossbreeding with Vil1-Cre mice (TMEM16F^fl/fl^-Vil1-Cre; KO) [31]. WT littermates (TMEM16F^fl/fl^; WT) served as controls.

### 4.9. Material and Statistical Analysis

Student’s *t* test for paired samples and ANOVA were used for statistical analysis. *p* < 0.05 was accepted as significant difference. HT29, MG63, A549, and Cal273 cells were from ATCC. All animal experiments were approved by the local Ethics Committee of the Government of Unterfranken/Würzburg (AZ: 55.2-2532-2-328) and were conducted according to the guidelines of the American Physiologic Society and German Law for the Welfare of Animals.

## 5. Conclusions

TMEM16A is overexpressed in a subset of malignomas where it drives cellular dedifferentiation, cell proliferation and thus supports growth of cancer cells [5]. A number of cellular signaling molecules appear to contribute to this pro-proliferative effect, however, TMEM16A-induced filling of the ER Ca^2+^ store and enhanced cytosolic Ca^2+^ signaling, appear to be major factors. While TMEM16A is overexpressed only in a subset of cancers, TMEM16F is expressed consistently at lower levels. Activation of TMEM16F supports different forms of regulated cell death. It is highly relevant for ferroptotic cell death, probably due to its plasma membrane destabilizing effect. Phospholipid scrambling and parallel activation of nonselective ion currents that leads to cell swelling, may support cell death. Thus, direct activation of TMEM16F in cancer cells is likely to kill cancer cells. The combined application with specific inhibitors of TMEM16A may reflect a novel powerful therapeutic strategy. This will require identification of paralog-specific activators and inhibitors.
